# Nanosheet BiOBr Modified Rock Wool Composites for High Efficient Oil/Water Separation and Simultaneous Dye Degradation by Activating Peroxymonosulfate

**DOI:** 10.3390/molecules29133185

**Published:** 2024-07-04

**Authors:** Li Lin, Si Xiao, Chuxuan Wang, Manhong Huang, Ling Xu, Yi Huang

**Affiliations:** 1 School of Material and Chemical Engineering, Hunan City University, Yiyang 413000, China; 2 Key Laboratory of Low Carbon and Environmental Functional Materials of College of Hunan Province, Yiyang 413000, China; 3 School of Environmental Science and Engineering, Donghua University, Shanghai 201620, China

**Keywords:** BiOBr, oil/water separation, peroxymonosulfate, dye removal, underliquid superlyophobic

## Abstract

The development of superlyophobic materials in liquid systems, enabling synchronous oil/water separation and dye removal from water, is highly desirable. In this study, we employed a novel superwetting array-like BiOBr nanosheets anchored on waste rock wool (RW) fibers through a simple neutralization alcoholysis method. The resulting BiOBr/RW fibers exhibited superoleophilic and superhydrophilic properties in air but demonstrated underwater superoleophobic and underoil superhydrophobic characteristics. Utilizing its dual superlyophobicity, the fiber layer demonstrated high separation efficiencies and flux velocity for oil/water mixtures by prewetting under a gravity-driven mechanism. Additionally, the novel BiOBr/RW fibers also exhibited excellent dual superlyophobicity and effective separation for immiscible oil/oil systems. Furthermore, the BiOBr/RW fibers could serve as a filter to continuously separate oil/water mixtures with high flux velocity and removal rates (>93.9%) for water-soluble dye rhodamine B (RhB) simultaneously by directly activating peroxymonosulfate (PMS) in cyclic experiments. More importantly, the mechanism of simultaneous oil/water separation and RhB degradation was proposed based on the reactive oxygen species (ROS) quenching experiments and electron paramagnetic resonance (EPR) analysis. Considering the simple modified process and the waste RW as raw material, this work may open up innovative, economical, and environmentally friendly avenues for the effective treatment of wastewater contaminated with oil and water-soluble pollutants.

## 1. Introduction

Oil spilling and industrial wastewater inflict serious damage on water ecosystems and pose a significant threat to human well-being through the food chain [1,2,3]. With the rapid increase of oil storage, transportation, and industrial wastewater discharge in recent decades, addressing oily wastewater containing grease and water-soluble organic pollutants has become an urgent necessity. Currently, in the pursuit of green processes with low energy consumption and high separation efficiency for separating oil/water mixtures, superwettability materials stand out as promising candidates for the quick and efficient removal of oil from marine/aquatic environments [4,5]. The unique superwettability primarily relies on regulating surface topography and chemical compositions. Among these superwettability materials, lotus leaf-inspired superoleophilic/superhydrophobic materials can only achieve heavy oil/water separation due to the barrier effect of the lower water layer, exposing them to the risk of oil contamination. Conversely, materials inspired by fish scales, exhibiting superhydrophilicity and underwater superoleophobicity, can effectively separate light oil and water by preventing oils from permeating through the water-locking layer. While this technique addresses oil pollutants, it does not solve the issues of wettability conversion and dissolved organic compounds.

To simultaneously tackle these problems, designing and developing multifunctional composite materials with reversible wetting properties that can both remove soluble organic contaminants and selectively separate oil/water mixtures is necessary and urgent. Zhang et al. reported a pH-responsive melamine sponge with switchable wettability and photocatalytic activity, demonstrating high efficiency for oil/water separation [6]. Gondal et al. [7] and Kwon et al. [8] reported a stainless steel membrane coated with rare-earth oxide or iron-doped titanium oxide nanoparticles with superhydrophilic characteristics. These membranes exhibited high efficiency in oil/water separation and enhanced photocatalytic activity for degrading persistent organic pollutants in water. Additionally, functionalized and surface-modified cotton fabrics have shown high efficiency in photocatalytic degradation of dissolved organic pollutants and separating oil from water [9,10,11]. Huang et al. [12] reported a superhydrophobic composite by grafting P25 nanoparticles and polydimethylsiloxane on alkaline-etched copper mesh (P25@PDMS@COM) with superhydrophobic, self-cleaning, photocatalytic characteristics suitable for oil/water separation. Even though surface modification and coating processes can improve the efficiency of materials for oil/water separation and degradation of water-soluble organic contaminants, they may lead to shortcomings such as high costs, energy consumption, complex equipment for light irradiation, and the introduction of new pollutants to the environment. Moreover, while various wetting strategies between underoil superhydrophobic (UO-SHB) and underwater superoleophobic (UW-SOB) surfaces have been achieved by regulating surface morphology and chemical properties, few of them have been applied to continuous on-demand oil/water separation due to the specific wettability conversion stimulus such as pH or temperature.

Recently, advanced oxidation processes (AOPs) based on peroxymonosulfate (PMS)/persulfate (PS) have been introduced in the oil/water separation to synchronously remove water-soluble organic contaminants in the absence of light. For instance, Gao et al. [13] developed a microalgae-based membrane equipped with a continuous reactor system, achieving excellent oil/water separation efficiency and outstanding removal efficiency of water-soluble organic contaminants in the presence of potassium persulfate (PS). Zhang et al. [14] fabricated a polyvinylidene fluoride/β-FeOOH (PVDF/β-FeOOH) catalytic membrane, realizing high-efficiency degradation of recalcitrant pollutants and high oil rejections simultaneously under low operating pressure in the presence of PMS. Additionally, CoFe_2_O_4_@PVDF membrane [15] and Ni/Co LDH stainless steel mesh [16] et al. have been reported as mulfunctional materials for oil/water separation and degradation of water-soluble organic pollutants via coupling with PMS activation. Compared to the photocatalytic degradation process, PMS/PS-based AOPs overcome the disadvantages, including insufficient contact between light and material and the need for extra light energy and lighting equipment. However, some reported PMS/PS-based catalytic membranes exhibited low flux velocity and unswitchable wettability toward the oil/water separation process. Therefore, developing environmentally friendly and cost-effective materials with high flux velocity and easily controllable wettability conversion for simultaneous effective oil/water separation and removal of organic contaminants remains a challenge to be addressed.

Rock wool (RW) is an inorganic fabric substance made from basalt or dolomite, widely used in various industrial applications, including sound absorption, fire and heat insulation, and cement reinforcement, owing to its low cost, high chemical stability, and incombustibility [17]. However, the disposal of RW waste can lead to economic disadvantages and environmental pollution issues, as it occupies a large footprint and undergoes a challenging biodegradation process. To address this problem, it is crucial to explore new processes for the possible recycling of waste RW. Hao et al. developed modified RW with excellent oil-absorbing capacity for separating high-viscosity oil through assisted electric heating [18]. Cai et al. utilized functionalized waste RW for the removal of Cr (VI) from contaminated water and soil [19]. Due to its hydrophobicity and high porosity, RW exhibits excellent oil/water separation characteristics, making it a promising and inexpensive adsorbent for various environmental restoration processes, such as oil spill accidents. While RW shows some oil-water separation properties, it lacks the capacity for on-demand oil/water separation and removing water-soluble organic contaminants. It is speculated that constructing specific micro-nano structures by anchoring a certain catalyst to modify the RW surface is expected to solve the above problems.

BiOX (X = I, Br, Cl) has gained significant attention recently due to its excellent photocatalytic properties, attributed to its suitable bandgap, strong photocatalytic oxidation, chemical stability, low cost, and eco-friendliness [20,21]. BiOBr and BiOI are especially known for their photocatalytic activity under visible light [22,23]. However, using them as fine powders for water pollutant removal poses several technical challenges, such as high density, large pressure drop, and difficulty in recycling/reusing due to their dispersed nature. Moreover, the light utilization efficiency is severely affected, as in the powdery TiO_2_ suspension system [24]. To address these technical issues, recent efforts have focused on loading BiOX onto the surface of different carriers/substrates, such as expanded graphite (EP) [25], fly ash cenospheres [26], and polyacrylonitrile nanofibers [14]. Notably, previous studies have indicated that Bi-based compounds possess the capability to directly activate peroxymonosulfate (PMS) or persulfate (PS) to remove soluble organic contaminants in the absence of light irradiation [27]. In addition, the unique nanosheet-like microstructures of BiOBr may endow some surfaces with superwettability. This provides inspiration to develop a multifunctional material by anchoring micro-nano BiOBr onto waste RW fibers using a facile method, aiming to handle oily wastewater containing water-soluble organic pollutants in complex environmental remediation projects.

In this project, superhydrophobic RW, an industrial solid waste, was utilized as a carrier/substrate for fabricating BiOBr nanostructures, creating a novel multifunctional material using a one-step neutralization alcoholysis modification method. The resulting multifunctional fibers exhibited superhydrophilicity and superoleophilicity in air and underliquid dual superlyophobic properties (UW-SOB and UO-SHB) due to the anchoring array-like BiOBr nanosheets. The oil/water separation capability and catalytic performances of the developed composites were thoroughly explored. Experimental results demonstrated that the BiOBr/RW multifunctional fibers exhibited outstanding capability for on-demand oil/water separation and the degradation of dyes in the presence of PMS. Moreover, the novel composite displayed excellent physicochemical stability and recyclability. This low-cost and eco-friendly multifunctional material holds great potential for large-scale applications in environmental remediation processes, especially in the removal of pollution caused by oil spill accidents.

## 2. Results and Discussion

### 2.1. Properties of Samples

**Figure 1:**
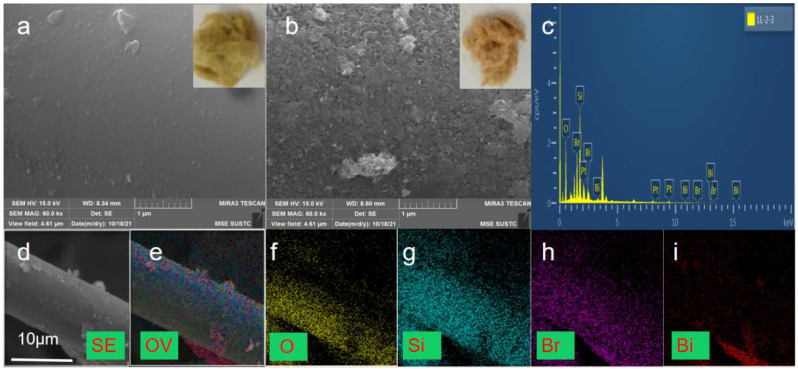
Digital photos and SEM images ((**a**) pristine RW; (**b**) BiOBr/RW), EDS spectra of BiOBr/RW (**c**), and EDS mapping images of BiOBr/RW (**d**–**i**).

The morphologies of the obtained samples were observed through SEM images and digital photos. Results are presented in Figure 1. RW and BiOBr/RW depicted in the inner illustrations of Figure 1a,b exhibit the composition of a large number of crisscrossing loose fibers. The cross-linked porous network structure, resembling a cotton-like morphology, indicates that BiOBr/RW has the potential to adsorb pollutants from oil/water mixtures. Additionally, compared to the inner illustrations of Figure 1a, the gradual deepening of the modified sample’s color, as shown in the inner illustrations of Figure 1b, transitioning from light yellow to brownish yellow, confirms the presence of a new substance loaded on the surface of RW. Comparing the relatively smooth surface of RW in Figure 1a, it is evident from Figure 1b that the relatively rough and irregular substance anchored onto the surface of RW fibers consists of a large number of array-like nanosheets. These micro-nano-structured nanosheets may be closely attached to the surface of the RW fibers, contributing to the observed roughness. This unique surface topography of BiOBr/RW fibers is beneficial for retaining liquids or air, endowing the surface with superwettability critical for its oil-water separation performance [28,29]. Figure 1c displays the EDS spectra of BiOBr/RW, indicating the element composition involved Bi, Br, Si, and O with the Bi:Br molar ratio approximately 1:1. Figure 1d indicates the diameter of modified RW is about 10 μm, and its corresponding EDS elemental mapping images in Figure 1e–i confirmed the composite constructed by anchoring BiOBr onto the pristine RW fiber surfaces.

**Figure 2:**
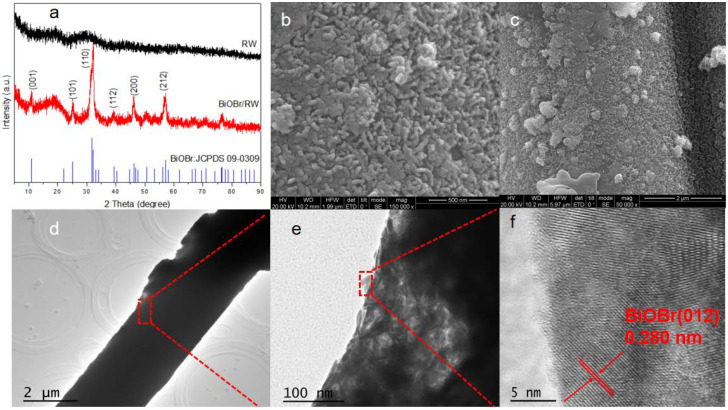
XRD patterns of the samples (**a**), HRSEM images of BiOBr/RW (**b**,**c**), and HRTEM images of BiOBr/RW (**d**–**f**).

To further confirm the anchoring of BiOBr on RW fibers, the crystal structures of both pristine and modified RW samples were characterized using XRD. As shown in Figure 2a, the broad diffraction peak at 29.5° indicates the presence of CaO-MgO-SiO_2_-Al_2_O_3_ compound in the pristine waste RW [30]. In contrast, the diffraction peaks of BiOBr/RW at 32.3° and 46.3° can be attributed to (110) and (200) planes, matching well with the tetragonal BiOBr (JCPDS 73-2061) [31]. The HRSEM images in Figure 2b,c further confirm the unique surface topography composed of array-like nanosheets with thicknesses of 20–50 nm. The HRTEM image in Figure 2f indicates continuous lattice fringes with an inter-planar distance of 0.280 nm, corresponding to the (012) plane of the BiOBr phase. These results confirm the successful anchoring of BiOBr on pristine RW [32].

### 2.2. Wettability and Separation Capacity

Surface wettability plays a crucial role in oily wastewater treatment and oil spill recovery. The wettability of the as-prepared BiOBr/RW and pristine RW was investigated under various environments. The tested contact angles are defined as oil contact angle in the air (CA_o_), water contact angle in the air (CA_w_), oil contact angle underwater (CA_o/w_), and water contact angle underoil (CA_w/o_).

**Figure 3:**
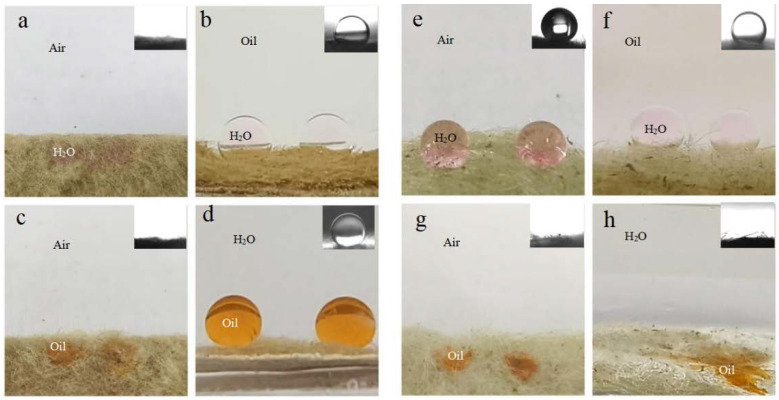
Photos of different liquid wetting behavior and corresponding contact angle (insets) under various environments on BiOBr/RW fibers (**a**–**d**) and pristine RW (**e**–**h**), respectively.

**Figure 4:**
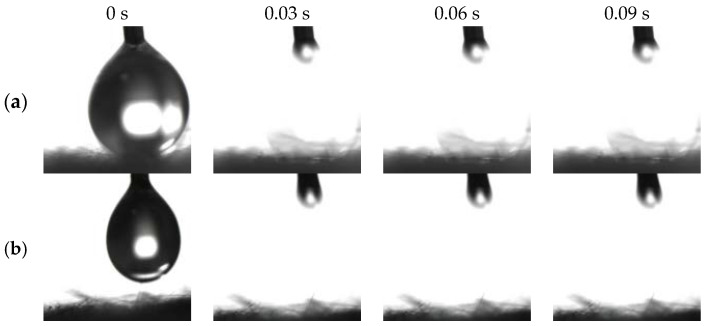
Photos of penetration process on BiOBr/RW surface in the air ((**a**) water droplet; (**b**): oil droplet).

Figure 3a,c exhibits distinct amphiphilicity of the as-prepared BiOBr/RW in air. In addition, the unique dual superlyophobicity under different liquid environments shown in Figure 3b,d was clearly observed on the surface of BiOBr/RW fibers, which exhibit both underoil superhydrophobicity (CA_w/o_ = 155.0°) and underwater superoleophobicity (CA_o/w_ = 155.3°). Figure 4 demonstrates that two types of liquid droplets rapidly permeated into the BiOBr/RW fiber layer within less than 0.09 s, further confirming the superhydrophilicity and superoleophilicity in air. Contrastly, as shown in Figure 3e,g, the pristine RW exhibits superhydrophobicity (CA_w_ = 152.7°) and superoleophilicity (CA_o_ = 0°) in air, corresponding to the water droplet maintaining spheroidal shape on the RW layer surface but the oil droplet rapidly spreading and penetrating into the RW fibers. Additionally, as shown in Figure 3f,h, the pristine RW maintains underoil superhydrophobicity (CA_w/o_ = 156.3°) and underwater superoleophilicity (CA_o/w_ = 0°). At the same time, the unique mirror effect underwater for hydrophobic materials can be clearly observed. The above results manifest that the pristine RW possesses superhydrophobicity and superoleophilicity, whether under liquid or in air. The significant differences in wettability between pristine RW and BiOBr/RW may have contributed to distinct oil/water separation performance.

**Figure 5:**
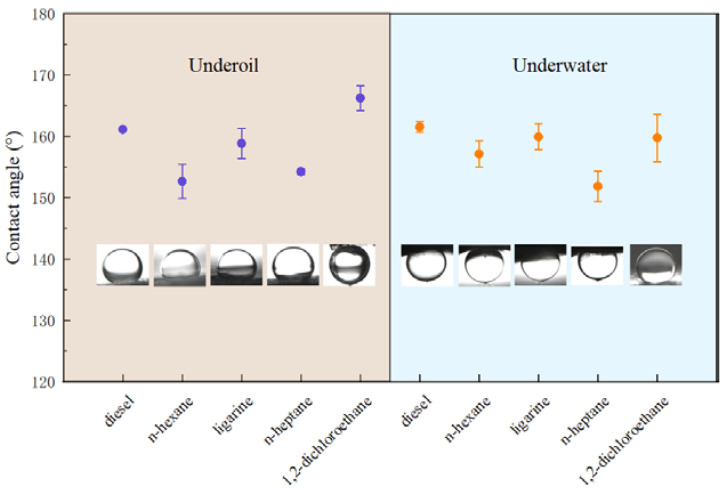
CA_o/w_ and CA_o/w_ of various oils detected on BiOBr/RW.

Generally, CA_o/w_ and CA_w/o_ sum up to 180° on a solid surface [33]. From a thermodynamic perspective, it is contradictory that these two properties are presented on the same surface simultaneously. Herein, the Cassie model [34,35] could be applied to explain the observed dual superlyophobic phenomenon. For instance, when the as-prepared BiOBr/RW is immersed into liquid 1 (oil/water), the liquid 1 molecules rapidly invade the unique roughness topography structures and occupy the gap space, in which the captured and formed liquid 1 layer will act as a repulsive liquid to hinder the penetration of liquid 2 and result in the underliquid superlyophobicity. Combined with the previous HRSEM analysis, the formed roughness topography via nanosheet BiOBr endows the RW fibers with superlyophobicity under oil/water liquids. This unique dual superlyophobic property is expected to endow BiOBr/RW with on-mand oil/water separation performances by switching oil/water wettability. The wettability of BiOBr/RW was further detected by various oils such as n-heptane, diesel, n-hexane, 1.2-dichloromethane, and ligarine. Figure 5 indicates that the corresponding CA_o/w_ are all larger than 150°, suggesting the surface has been bestowed with outstanding underliquid superlyophobicity.

**Figure 6:**
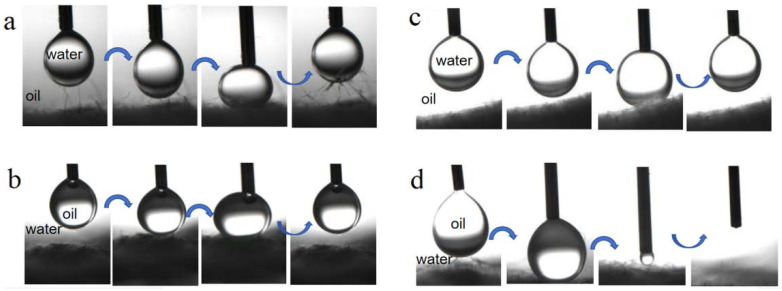
Dynamic interaction of water droplet underoil (ligarine) and oil (1,2-dichloroethane) droplet underwater on the surface of BiOBr/RW (**a**,**b**) and pristine RW (**c**,**d**) immersed in oil or water.

Figure 6 illustrates the dynamic interaction process revealing the underwater oil antifouling and underoil water antifouling of both BiOBr/RW and pristine RW. In a preloading-relaxing cycle, a suspended droplet of oil and water was directed to contact the surface of the fibers. As depicted in Figure 6a,c, the water droplet easily lifted off instead of adhering to the surfaces of both BiOBr/RW and pristine RW when the fibers were immersed in oil (ligarine). A similar observation in Figure 6b indicated that the oil droplet (1.2-dichloromethane) remained at the tip of the needles without adhering to the surface of BiOBr/RW fibers during the same cycle when immersed underwater. Differently, Figure 6d demonstrates the complete penetration of the oil droplet into the RW fiber layer, confirming the underwater superoleophilicity. These results confirmed that the as-prepared BiOBr/RW possesses excellent underliquid antifouling properties.

**Figure 7:**
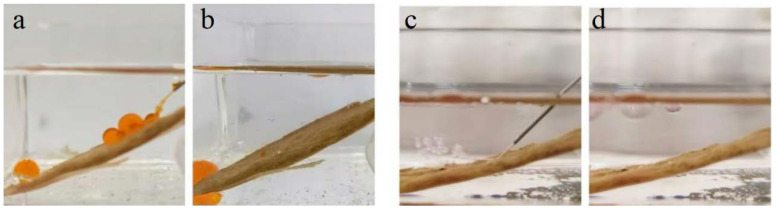
A dynamic liquid jet process on BiOBr/RW. Oil (Sudan red I dyed 1,2-dichloroethane) jet underwater (**a**,**b**) and water jet underoil (1,2-dichloroethane) (**c**,**d**).

In addition, a dynamic liquid jet test was conducted on the surface of BiOBr/RW fibers. As depicted in Figure 7a,b, the jetted oil (1.2-dichloromethane) rapidly formed spherical shapes and rolled to the bottom, demonstrating its inability to adhere to the surface of the BiOBr/RW fiber layer underwater environment. Simultaneously, the jetted water column rebounded and floated up to the oil (1.2-dichloromethane) surface without leaving a trace after being sprayed onto the BiOBr/RW fiber layer surface (Figure 7c,d). These results provide further evidence that the stable pre-wetting water/oil layers existed on the surface of BiOBr/RW, serving as a repulsive liquid phase that prevents the counterpart phase from adhering to the fiber layer’s surface. Therefore, BiOBr/RW fibers exhibit low adhesion and outstanding antifouling properties underliquid, showcasing their dual-superlyophobic wettability.

**Figure 8:**
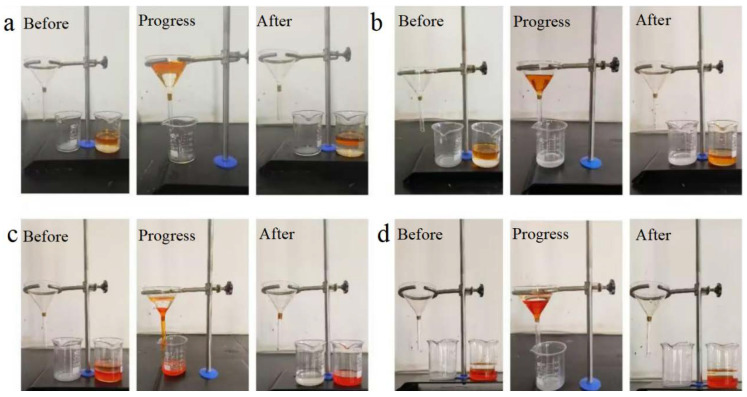
Images of oil/water separation by BiOBr/RW ((**a**) prewetted by light oil; (**b**) prewetted by water; (**c**) prewetted by heavy oil; (**d**) prewetted by water); light oil and heavy oil were dyed with Sudan I.

Based on the dual-superlyophobic wettability, the as-prepared BiOBr/RW fibers are expected to possess unique capabilities in selectively separating oil and water. To assess this property, a homemade filter apparatus with a triangular funnel was utilized, as illustrated in Figure 8. Generally, the apparent stratification phenomenon led to the water layer consistently residing above the oil layer in light oil/water mixtures and beneath the oil layer in heavy oil/water scenarios. For light oil/water mixture separation, when the BiOBr/RW fibers were prewetted with light oil (Figure 8a), water was prevented from flowing through the fibers, resulting in the light oil layer being retained above the water layer. Conversely, in Figure 8b, when the modified RW fibers were prewetted with water, water rapidly traversed the fibers into the bottom beaker, while the light oil was confined in the upper receiver situated above the water-prewetted fibers. The separated light oil in the funnel could then be effortlessly collected. A similar procedure was observed for heavy oil/water mixture separation in Figure 8c. In this case, the heavy oil swiftly traversed the fibers to the beaker when the BiOBr/RW fibers were prewetted with oil, leaving water on the oil-prewetted BiOBr/RW fibers. However, this separation process was unattainable if the BiOBr/RW fibers were prewetted with water, as shown in Figure 8d. Due to the underliquid dual superlyophobicity, prewetting with water caused the BiOBr/RW fibers to be saturated with water. This saturation led to the trapping of the porous structure, creating a water layer that could impede the passage of oil through the BiOBr/RW layer [36]. Conversely, when prewetted with the oil phase, BiOBr/RW absorbed and retained oil, forming an oil layer that prevented water droplets from permeating the BiOBr/RW layer. The previous SEM and XRD analyses revealed that the pristine RW’s smooth surface underwent a transformation into roughness after anchored array-like BiOBr nanosheets. This alteration caused Cassie’s model to dominate, ultimately resulting in the performance of switchable oil/water separation [37].

**Figure 9:**
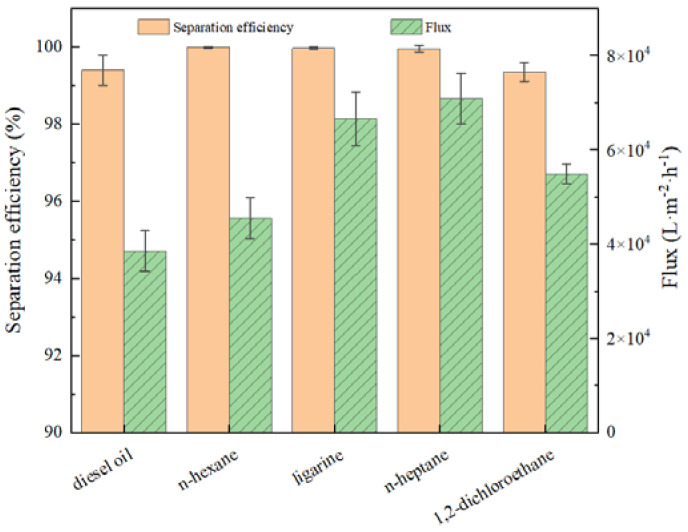
The separation efficiency and flux for different oil/water mixtures.

To investigate the separation efficiency and flux toward different oil/water mixtures by prewetted BiOBr/RW, some experiments were conducted. As depicted in Figure 9, the BiOBr/RW demonstrated the separation efficiencies all exceeding 99.9% for different water/light oil (n-hexane, ligarine, or n-heptane) mixtures when prewetted with water. No organic substances were detected in the filtrates, indicating the high purity of the separated water. The slightly lower separation efficiency (approximately 99.4%) observed for diesel may be attributed to the volatilization loss. Moreover, the separation efficiency for water/heavy oil (1.2-dichloromethane) surpassed 99.4%, with no water droplets detected in the filtrates when the fibers were prewetted with oil. The water flux in the water/light oil separation process exceeded 30,000 L·m^−2^·h^−1^, reaching a maximum of 70,891.3 L·m^−2^·h^−1^ corresponding to the water/n-heptane system. The oil flux for water/heavy oil (1.2-dichloromethane) separation was measured at 54,906.6 L·m^−2^·h^−1^. These results demonstrated excellent separation efficiency and higher flux than most of the reported membranes.

**Figure 10:**
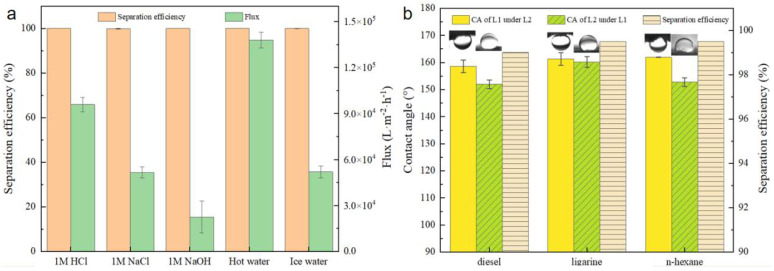
Separation efficiency and flux of ligarine/water mixtures under different environments (**a**), the separation efficiency and contact angles detected by various immiscible liquids mixtures (Liquid 1 is diesel, n-heptane or ligarine; Liquid 2 is EG) (**b**).

Additionally, Figure 10a illustrates the stability of BiOBr/RW when utilized to separate challenging oil/water mixtures containing ligarine/1 M water solution (HCl aq, NaCl aq, or NaOH aq), hot water (80 °C), and cold water (4 °C). The separation efficiencies of BiOBr/RW maintained consistently above 99.9%, highlighting its robust performance across various harsh conditions. The water flux exhibited a decrease trend with an increase of alkalinity in the mixture. However, higher water temperatures were observed to enhance the water flux during the separation process. Notably, the underoil superhydrophobicity of BiOBr/RW contributed to the effective hindrance of external ions, preserving the integrity of the fiber surface and emphasizing its stability.

In addition to its application in oil/water mixtures, BiOBr/RW demonstrated effective separation of immiscible organic solvents. Figure 10b depicts contact angles of EG measured under diesel, ligarine, and n-hexane, all exceeding 150°. Similarly, the contact angles of diesel, ligarine, and n-hexane under EG were also higher than 150°. This dual superlyophobicity under immiscible organic solvents can be well explained by Cassie’s mechanism. According to this mechanism, liquid 1/liquid 2 would rapidly permeate and be captured by the unique topography constructed by the surface BiOBr nanosheets. The trapped liquid then acts as a repulsive layer, preventing the invasion of the other immiscible liquid. Therefore, the separation of immiscible organic solvents can be achieved by prewetting the BiOBr/RW fiber layer. The separation efficiencies exceeding 99.5%, as shown in Figure 10b, indicate that BiOBr/RW fibers excel in immiscible solvent separation, holding great promise for treating organic solvents.

### 2.3. Removal Performance for Water-Soluble Pollutant

**Figure 11:**
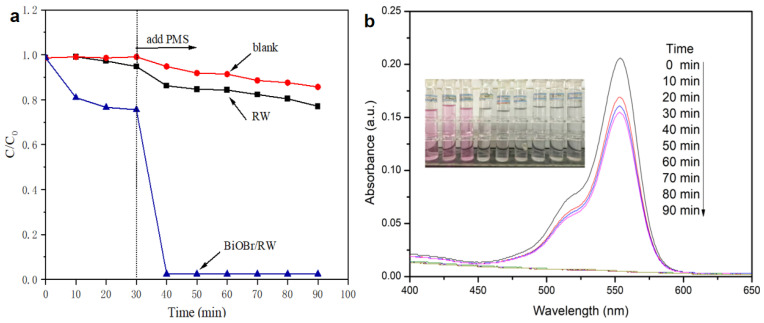
Degradation of RhB in direct PMS activation system by BiOBr/RW (**a**), and the change of UV-vis spectra with time for RhB degradation in BiOBr/RW/PMS system (**b**).

A variety of water-soluble organic pollutants, including dyes, are often present in oily wastewater from industrial releases. Therefore, the effective degradation of water-soluble organics in the oil/water separation process is crucial for safeguarding water environments. In this study, RhB was utilized as a soluble organic pollutant to assess the degradation activities of BiOBr/RW through direct PMS activation in the dark. As illustrated in Figure 11a, only 14.3% of RhB was removed in the presence of PMS alone, indicating the weak self-decomposition properties of PMS. The removal efficiency of RhB increased to 22.9% after the addition of pristine RW, suggesting that RW alone cannot significantly activate PMS to degrade RhB. However, upon the addition of BiOBr/RW, the fibers were readily immersed into the RhB solution, and 23.4% of RhB was removed by adsorption when an adsorption-desorption equilibrium was achieved within 30 min in the dark. Subsequently, when PMS was added into the mixture system, the RhB concentration decreased rapidly, and 97.6% of RhB was removed in the first 10 min. The initial fast degrading trend slowed down as the reaction progressed, likely due to the consumption of PMS. The immediate decolorization and diminished adsorption peak at approximately 554 nm (Figure 11b) further confirmed the rapid degradation of RhB. Compared with the pristine RW, the enhanced performance toward PMS direct activation is mainly attributed to the anchored BiOBr nanosheets on the RW fiber surface, enhancing its roughness, specific surface area, and hydrophilicity, further facilitating the contact between RhB and surface active sites. In addition, the reactive redox species generated via PMS direct activation by BiOBr/RW play a key role in the effective removal of RhB. This direct activation of PMS to degrade RhB aligns with previously reported results [14]. Therefore, owing to their superlyophobic and unique PMS-based catalytic properties, BiOBr/RW fibers present a promising material for achieving oil/water separation and the simultaneous degradation of organically soluble pollutants.

**Figure 12:**
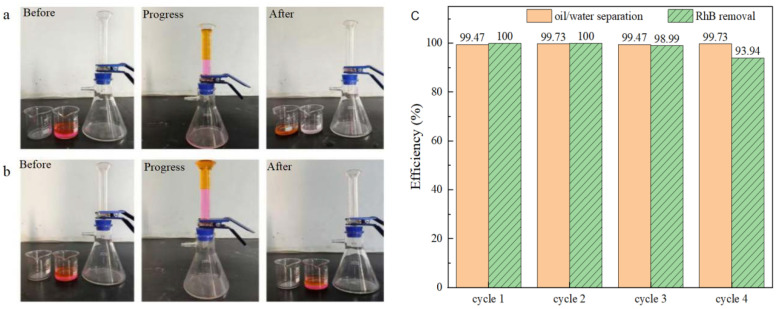
The synchronous oil/water separation and PMS activation toward RhB removal ((**a**) BiOBr/RW; (**b**) pristine RW; (**c**) cycle results).

To assess the performance of simultaneous oil/water separation and PMS activation for the degradation of water-soluble organic contaminants, tests were conducted over four cycles using BiOBr/RW to treat simulated complex wastewater containing both oil and water-soluble dye pollutants under identical conditions. The simulated complex wastewater comprised 25 mL of water (RhB: 5 mg/L; PMS: 200 mg/L) and 25 mL of light oil ligarine. The experiments were conducted using a triangular bottle filtration device, with 0.3 g of BiOBr/RW placed at the bottom of a graduated glass tube. As depicted in Figure 12a, when the complex wastewater was poured into the separator, the color of the filtrate noticeably changed from rose-red to transparent in each cycle, while the light oil was effectively blocked by the water-prewetted BiOBr/RW layer. In contrast, as shown in Figure 12b, pristine RW failed to achieve RhB removal and light oil/water separation under the same conditions. Previous results suggest that RhB dye removal can be attributed to the synergistic effect of superhydrophilicity, adsorption, and degradation by activated PMS. Figure 12c indicates that the efficiency of oil/water separation and RhB removal remained consistently above 99.4% and 94.0%, respectively, after four cycles, indicating the remarkable reusability of BiOBr/RW. These findings imply that the reusable BiOBr/RW fiber can overcome the recycling challenges associated with traditional powdery catalysts. Moreover, synchronous oil/water separation and water-soluble organic contaminant removal through PMS activation can be achieved by altering the feeding method and adjusting the amount of catalyst filled in the columnar reactor. Therefore, BiOBr/RW demonstrates great potential as a promising multifunctional material for oil/water separation and the simultaneous degradation of soluble organic pollutants.

**Figure 13:**
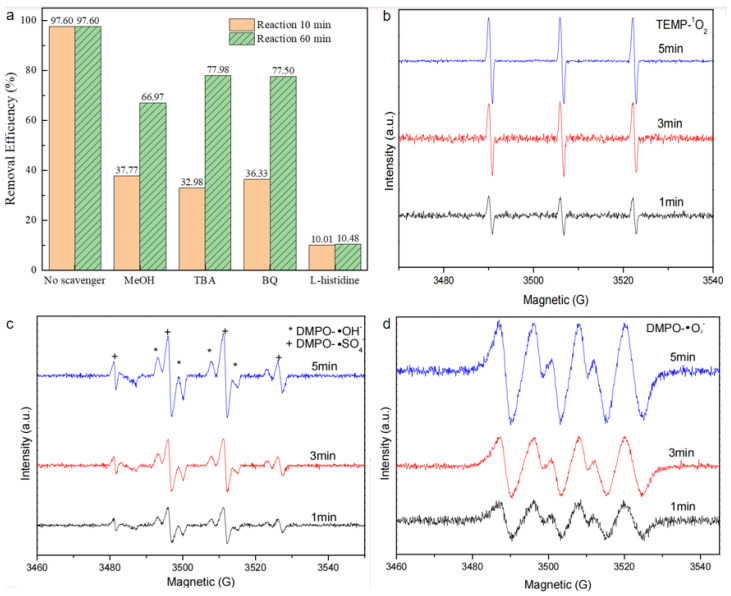
Quenching experiments results in the BiOBr/RW catalyst/PMS direct activation system (**a**) and ESR spectra in different systems: (**b**) ^1^O_2_, (**c**) ·SO_4_^−^ and ·OH, and (**d**)·O_2_^−^.

To elucidate the mechanism of synchronous RhB removal, quenching experiments were conducted to detect the participation of reactive oxygen species in the BiOBr/RW/PMS system. In these experiments, MeOH, BQ, TBA, and L-His were selected as quenchers for ·SO_4_^−^/·OH, ·O_2_^−^, ·OH, and ^1^O_2_, respectively [38]. As depicted in Figure 13a, all added quenchers exhibited distinct inhibitory effects on RhB degradation after the addition of PMS. The removal rates of RhB decreased from 97.6% to 37.8%, 33.0%, 36.3%, and 10.0% in the first 10 min, corresponding to MeOH, TBA, BQ, and L-His, respectively. Eventually, the RhB degradation rate was inhibited following the addition in the order of L-his, MeOH, TBA, and BQ, with the corresponding removal rates achieving 10.5%, 67.0%, 78.0%, and 77.5% in 90 min, respectively. This result suggests that ^1^O_2_ was the primary oxidant. Furthermore, ·SO_4_^−^, ·OH, and ·O_2_^−^ all contributed to the degradation process of RhB. Additionally, the EPR test was used to verify the reactive species further. As shown in Figure 13b, when 2,2,6,6-tetramethyl-4 -piperidinol (TEMP) was used as the spin-trapping agen of ^1^O_2_, a significant 1:1:1 triple signal of TEMP-^1^O_2_ was observed clearly, indicating that the formation of ^1^O_2_ from PMS activation [39]. To confirm whether ·SO_4_^−^, ·OH, and ·O_2_^−^were produced, 5,5-dimethyl-1-pyrroline N-oxide (DMPO) was used as the spin-trapping agent [40]. As shown in Figure 13c, a strong characteristic 1:2:2:1 peak signal could be attributed to the formation of DMPO-·OH. This was consistent with the results of the previous trapping experiment. In addition, a characteristic signal corresponding to DMPO-·O_2_^−^ was observed in Figure 13d, suggesting ·O_2_^−^ participated in the direct PMS activation process by BiOBr/RW [41]. All these observed signal intensities were increased with the time prolonged in the following 5 min. Therefore, it can be concluded the ^1^O_2_, ·SO_4_^−^, ·OH, and ·O_2_^−^ radicals were presented in the BiOBr/RW/PMS system. Therefore, the generated ^1^O_2_, ·SO_4_^−^, ·OH and ·O_2_^−^ all participated the RhB degradation.

**Figure 14:**
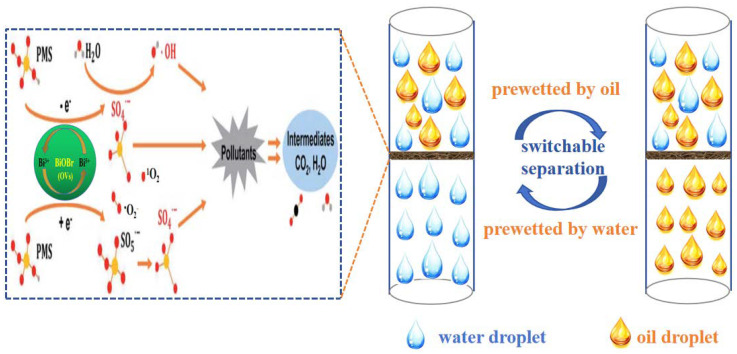
Mechanism of synchronous on-demand oil/water separation and PMS activation for pollutants removal by BiOBr/RW.

According to the above results, a suggested schematic mechanism for the treatment of complex wastewater via synchronous oil/water separation and direct PMS activation for the removal of water-soluble organic contaminants is illustrated in Figure 14. Based on dual superlyophobicity, the BiOBr/RW fiber layer can selectively block the passage of oil or water by pre-wetting, achieving on-demand oil/water separation. Moreover, whether light oil/water mixture or heavy oil/water mixture, the complex wastewater can be made to pass through the BiOBr/RW fiber layer by employing different feeding methods, such as from top to bottom or from bottom to top. Consequently, the water-soluble organic pollutants can react with the produced radicals and be degraded in the presence of PMS simultaneously. Combining capturing experiments and EPR results, a possible mechanism for the degradation of water-soluble organic pollutants through the direct activation of PMS is proposed and illustrated in Figure 14. Firstly, the induced Bi^3+^-Bi^5+^-Bi^3+^ redox cycle, promoted by PMS activation, leads to the generation of nonradical oxidation (^1^O_2_) (Equation (1)) and ·OH/·SO_4_^−^ (Equations (2)–(4)), playing a prominent role in the degradation of water-soluble organic pollutants [42,43]. Furthermore, a possible nonradical process involving the donation of electrons to oxygen vacancies (OVs) from the PMS reductant may accelerate the cycling of O_2_ and further promote the transformation product of ·O_2_^−^ (Equation (5)) [43,44,45]. Ultimately, the generated amounts of ^1^O_2_, ·OH, ·SO_4_^−^ and ·O_2_^−^ participate in the pollutants degradation reaction (Equation (6)). In summary, the unique wettability and excellent PMS-activated degradability of BiOBr/RW fibers contributed to the effective oil/water separation and RhB removal synchronously.

BiOBr/RW + HSO_5_^−^ → ^1^O_2_ (nonradical pathway) (1)

Bi(III) +2HSO_5_^−^ → Bi(V) +2·OH + 2SO_4_^2−^ (2)

Bi(III) +2HSO_5_^−^ → Bi(V) + 2·SO_4_^−^ + 2OH^−^ (3)

Bi(V) + 2HSO_5_^−^ → Bi(III) +2·SO_5_^−^ + 2H^+^ (4)

O_2_ + e^−^(OVs) →·O_2_^−^ (5)

^1^O_2_/·OH/·SO_4_^−^/·O_2_^−^ + pollutants → Production (6)

## 3. Experimental Section

### 3.1. Chemicals and Materials

Bi(NO_3_)_3_·5H_2_O, KBr, ligarine, n-heptane, C_2_H_4_Cl_2_, ethylene glycol (EG), n-hexane, ammonia, peroxymonosulfate (K_2_SO_4_·KHSO_4_·2KHSO_5_), methanol (MeOH), tert-butanol (TBA), p-benzoquinone (BQ), L-histidine (L-His) and rhodamine B (RhB) were analytical reagent purchased from Shanghai Aladdin Biochemical Technology Co., Ltd. (Shanghai, China). Diesel oil was obtained from a local gas station. Waste rock wool (RW) was provided by Hunan Jinye Environmental Technology Co., Ltd. (Chenzhou, China). All chemicals were used as received without further purification. Deionized water was obtained through a distilled water device in the lab.

### 3.2. Preparation of Samples

Typically, 2.8 mmol of Bi(NO_3_)_3_·5H_2_O was dissolved in 20 mL of EG solution with ultrasonication at room temperature (25 °C). Then, 2.8 mmol of KBr was dispersed and dissolved in the above solution by sonication for 30 min. RW (1.0 g) was added to the mixture and soaked for 30 min. Subsequently, the pH of the mixture was regulated to 7.0 by adding 25.0 wt% ammonia. The system was stirred continuously for 30 min, and the blend transformed into a suspension exhibiting a milky white appearance. The obtained precipitate underwent filtration and was subjected to heat treatment at 160 °C in an oven for 12 h. Subsequently, the heat-treated product underwent multiple washes with deionized water and was dried at 90 °C for 6 h.

### 3.3. Characterization Techniques

The morphology of BiOBr/RW and RW were observed using a field emission scanning electron microscopy (SEM) system (Tescan MIRA3, Brno, Czech Republic) equipped with energy-dispersive spectroscopy (EDS, Oxford AZtec, UK) and a high-resolution scanning electron microscopy (HRSEM) system (FEI, quanta400, Eindhoven, The Netherlands). The high-resolution transmission electron microscopy (HRTEM) images of the sample were obtained using a field emission transmission electron microscope (JEOL JEM-2100, Tokyo, Japan). The crystalline structures of BiOBr/RW and pristine RW were detected by X-ray diffraction (XRD) (Rigaku D/max-2550 diffractometer, Tokyo, Japan) using Cu-Kα radiation at an accelerating current of 200 mA and voltage of 40 kV. The samples were scanned in the 2θ range of 5° to 80°. The wetting behaviors of the prepared fibers were detected by a JC2000C1 contact angle measuring instrument (Zhongchen Digital Technology Co., Shanghai, China), and the average contact angle value was obtained by measuring at three different locations.

### 3.4. Oil/Water Separation Performance

The prepared sample was affixed to a simple homemade filtration device using a glass funnel. Subsequently, 50 mL of various mixtures consisting of a 1:1 volume ratio of oil/water was poured into the glass funnel and permitted to traverse the water-prewetted or oil-prewetted BiOBr/RW layer by gravity-driven solely. For observation, the oil and water were dyed with Sudan I and RhB, respectively. Separation efficiency was calculated as follows (7):

η = (V_1_/V_0_) × 100% (7)

Herein, V_1_ represents the volume of collected filtrate oil or water in the lower receivers, and V_0_ represents the volume of the original mixtures, respectively. The flux velocity was then determined based on V_1_ and the time (t/h) taken for the filtration process to complete. The cross-section area of the sample in contact with the oil was measured to be 0.785 cm^2^ (S/m^2^). The calculation formula is as follows (8):

Flux velocity = V_1_/St (8)

### 3.5. RhB Catalytic Degradation

To evaluate the catalytic properties of BiOBr/RW samples, RhB was employed as a representative organic contaminant. Firstly, 0.1g of BiOBr/RW sample was added to a RhB solution (50 mL, 10 mg/L) and allowed to reach equilibrium in darkness for 30 min. Secondly, the RhB degradation experiments via directly activating PMS were performed in the dark with the addition of 0.1 g/L PMS. During the reaction process, 1 mL of the sample was taken at given time intervals and diluted into a 10 mL colorimetric tube. The concentration of RhB was detected using the UV-vis spectrophotometer technique.

## 4. Conclusions

BiOBr/RW was successfully prepared via a facile neutralization alcoholysis process by utilizing waste RW as a carrier. The distinctive dual superlyophobic properties of BiOBr/RW are attributed to anchoring array-like BiOBr nanosheets onto the surface of RW. Comparative to pristine RW, BiOBr/RW exhibited the capability to separate oil/water mixtures on-demand by prewetting with an oil or water phase. Moreover, BiOBr/RW fibers demonstrated dual superoleophobicity in an immiscible oil/oil mixture, bestowing upon it unique oil/oil separation properties. Furthermore, the modified RWs displayed the capabilities for RhB removal by direct PMS activation synchronously in the cyclic oil/water separation processes. More importantly, based on the capturing experiments and EPR analysis results, all these radicals, such as ^1^O_2_, ·SO_4_^−^, ·OH, and ·O_2_^−^, were confirmed to participate in the RhB degradation. This study provides an efficient and environmentally friendly approach to making multifunctional purification materials from repurposing RW solid waste, indicating potential applications in mitigating complex water pollution resulting from oil spillage accidents, oily wastewater, and water-soluble organic pollutants discharge.

## Data Availability

Data are contained within the article.
